# 

**Published:** 1892-12

**Authors:** 


### TABLE OF CONTENTS.

### ORIGINAL COMMUNICATIONS.

PAGE

Treatment of Fracture of the Maxillæ. By Dr. William Carr, New York . . . 849

Crown- and Bridge-Work. (*Continued.*) By Dr. C. M. Richmond, New York . . . 858

Mechanics in Dentistry.—Is Dentistry a Science or a Profession? By W. G. A. Bonwill, D.D.S., Philadelphia . . . 860

Operative Failures and their Remedies. By Edwin C. Blaisdell, D.M.D., Portsmouth, N. H. . . . 868

The Dental Aspect of It. By A. Bethune Patterson, M.D. . . . 873

The Law of Heritage in Regard to the Teeth and Jaws. By Henry S. Chase, M.D., D.D.S., St. Louis, Mo. . . . 877

### REPORTS OF SOCIETIES.

New York Odontological Society . . . 880

American Dental Association . . . 884

American Academy of Dental Science . . . 895

Odontological Society of Pennsylvania . . . 898

Harvard Odontological Society . . . 903

### EDITORIAL.

Dentition: A Reply to Medical Criticism . . . 908

The Close of the Volume . . . 912

### CURRENT NEWS.

The World's Columbian Dental Congress, to be held in Chicago, August 17 to 27, inclusive, 1893 . . . 914

Recent Patents . . . 916

American Dental Society of Europe . . . 916

California State Board of Dental Examiners . . . 917

California State Dental Association . . . 917

### SELECTIONS.

Dentition in Infants . . . 917

Professor Harris . . . 920

Jaws of Inebriates . . . 921

Death under Pental . . . 922

Examination of Some Commercial Varieties of Hydrogen Dioxide . . . 922

Chemistry of the Cholera Bacillus . . . 923

